# Water distribution and key aroma compounds in the process of beef roasting

**DOI:** 10.3389/fnut.2022.978622

**Published:** 2022-09-13

**Authors:** Yong-Rui Wang, Rui-Ming Luo, Song-Lei Wang

**Affiliations:** ^1^College of Agriculture, Ningxia University, Yinchuan, China; ^2^College of Food and Wine, Ningxia University, Yinchuan, China

**Keywords:** water distribution, key aroma compounds, beef, roasting, correlation analysis

## Abstract

The key aroma compounds and water distribution of the beef at different roasting times (0, 3, 6, 9, 12, 15, and 18 min) were identified and analyzed. The results showed that the L^*^ value increased considerably before peaking and then decreased. On average, a^*^ values decreased significantly first and then kept stable, while b^*^ values increased first and then decreased. A total of 47 odorants were identified in all samples, including 14 alcohols, 18 aldehydes, 6 ketones, 1 ester, 3 acids, 4 heterocyclic compounds, and 1 other compound. Among them, 11 key aroma compounds were selected and aldehydes and alcohols predominantly contributed to the key aroma compounds. The fluidity of the water in the beef during the roasting process was decreased, and the water with a high degree of freedom migrated to the water with a low degree of freedom. The correlation analysis showed that water content and L^*^ were negatively correlated with key aroma compounds of the samples, while *M*_21_ was positively correlated with key aroma compounds.

## Introduction

Beef is widely consumed and has gained favor among many consumers worldwide because of its high-quality protein and vital elements such as essential amino acids, unsaturated fatty acids, minerals and vitamins (1). In 2019, China produced 6.67 million tons of beef, while imports were 1.6595 million tons and exports totaled just 0.02 million tons (2). *Qinchuan* cattle are the dominant breed in the Shaanxi-Gansu-Ningxia region. In the past 5 years, there have been about 3 million cattle in stock, with an average annual output of ~1 million cattle.

Aroma is a significant indication for evaluating food quality as well as a factor that influences consumer purchase behavior. Aroma compounds contribute to the aroma profile of meat and remarkably influence flavor perception (3). Raw meat has almost no aroma, and the majority of aroma compounds in meat were formed during the heating process. The primary reaction involved in the formation of aroma compounds in meat during heat processing includes lipid oxidation, Maillard reaction and Steckler degradation reaction, the interaction of lipids, Maillard reaction and thiamine degradation (4). Due to intramuscular lipid, many aroma compounds were identified at high concentrations even in lean muscle (5). Some linear aldehydes, alcohols, ketones and acid compounds were regarded as the byproducts of lipid oxidation (6). The Maillard process was principally responsible for the production of heterocyclic compounds such as pyrazines and furans. Multiple reactions arising from thermal degradation and lipid oxidation result in the formation of aroma compounds from non-volatile water-soluble precursors and lipids (5). Recently, a study evaluated 332 odorants found in thermally cooked meat by GC-O in the last 40 years by a search of relevant literature (7). A pleasant flavor is crucial for the appreciation of roasted beef. However, numerous compounds that are harmful to human health, such as heterocyclic amines (8), polycyclic aromatic hydrocarbons (9) and various kinds of aldehydes (10), were produced during the roasting process. Thus, understanding the production and key control points of aroma compounds during the roasting process of beef would help producers improve the aroma of beef or minimize the formation of unwanted odors (11).

Low field-nuclear magnetic resonance (LF-NMR) technology has been widely used in the analysis of the status, content and movement of water in samples as a standard instrument analysis method (12). It is most commonly employed in the food and agriculture fields since it is accurate, rapid and non-destructive (13–17). LF-NMR technology was also used to study the drying rate and textural features of meat products during drying (18). In addition, dynamic investigations have also been performed on meat undergoing processing including curing (19), cooking (20) and freezer storage (16). Meat contains considerable amounts of water (~70–75%), and the properties of the water are crucial for meat quality. The molecular mobility of water, protein and fat may affect the quality of the meat product during the heating process. The degree and nature of specific interactions (chemical or non-chemical, such as hydrogen bonding) between tiny solutes such as water molecules and ions and big food molecules may affect the meat's texture and flavor (21). In addition, water loss also means a reduction in production yield and thereby economic loss. Thus, the water in meat is important for both consumers and the meat industry.

The study aimed to (i) analyze and identify the aroma compounds of roasted beef during the electric roasting process through gas chromatography-mass spectrometry (GC-MS) combined with chemometric analysis; (ii) determine the key aroma compounds in roasted beef during the electric roasting process based on odor activity values (OAVs); (iii) analyze the water distribution and migration in the roasted beef for different periods by LF-NMR; (iv) determine the correlation between the moisture and color value and the formation of key aroma compounds in roasted beef The study may provide useful information and guidance for proper selection of roasting processes in the production of industrial beef.

## Materials and methods

### Materials

Beef from the hind legs of the *Qinchuan* cow was purchased from Yichuan's Xinbai market (Ningxia, China). The following chemicals were purchased from Sigma-Aldrich (Shanghai, China):1,2-dichlorobenzene (internal standard) and n-alkanes (C_7_-C_40_, ≥ 97%), hexanal (95%), heptanal (97%), 1-heptanol (97%), octanal (99%), (*E*)-2-octenal (97%), non-anal (99.5%), (*E*)-2-non-enal (97%), benzaldehyde (99.5%), (*E, E*)-2,4-decadienal (94%), 2-pentylfuran (98%), 2,5-dimethyl pyrazine (98%) and 1-octen-3-ol (98%). (*E*)-2-undecenal (l (94%), 2-pentylfuran (98%), 2,5-dime. (*E, E*)-2,4-non-adienal (95.8%) was from TCI Development Co., Ltd. (Shanghai, China). (*E*)-2-hexenal (97%) and (*E*)-2-heptenal (97%) were purchased from TCI (Shanghai, China). Methanol (analytical grade) was purchased from Thermo Fisher Scientific Co., Ltd (Shanghai, China).

### Sample preparation

The lean meat of the *Qinchuan* cow was separated and cut into 3.0 × 3.0 × 2.0 cm slices after the fascia was removed. The beef was then placed in an HQ-405 electric oven (Qingdao Hanshang Electric Company, Ltd., Shandong, China) for roasting with both top and bottom burners set to 250°C. The samples were roasted for a total of 0, 3, 6, 9, 12, 15, and 18 min and three replicates were performed for each sample.

### Instrumental color analysis

The color (CIE-L, a, b) of samples was determined using a TES-135A Chroma Meter (TES Co., Ltd., Taiwan, China). After roasting, the samples were placed in the room for 20 min to allow the roasted beef at the same temperature as room temperature (25 ± 1°C). Prior to measurement, the instrument was calibrated using a standard white plate covered with white paper (22). Meat samples were placed on a white paper and the color of the meat's surface was measured. L^*^ represents the lightness component, with a value ranging from 0 to 100 (from black to white). a^*^ denotes a red-green chromatic component with a value ranging from −60 to +60 (from green to red). b^*^ is a yellow-blue chromatic component with a range from −60 to +60 (from blue to yellow). Total color difference (ΔE) was calculated according to Eq. (1).


(1) $$\begin{array}{l} \Delta E=\sqrt{{\left(L^{{\;}^{*}}-L_{0}^{{\;}^{*}}\right)}^{2}+{\left(a^{{\;}^{*}}-a_{0}^{{\;}^{*}}\right)}^{2}+{\left(b^{{\;}^{*}}-b_{0}^{{\;}^{*}}\right)}^{2}} \end{array}$$

where ΔE is the color difference between raw beef and roasted samples; $L_{0}^{*}$, $a_{0}^{*}$, $b_{0}^{*}$ and L^*^, a^*^, b^*^ are the color parameters of the raw and roasted beef, respectively.

### Analysis of aroma compounds

#### GC-MS

Aroma compounds were analyzed by a GC-MS system (GC-MS 2010 plus, SHIMADZU) equipped with a DB-WAX capillary column (30 m × 0.25 mm × 0.25 μm, Agilent Technologies, Santa Clara, CA). The SPME fiber of 50/30 m DVB / CAR / PDMS should be aged before extracting the aroma compounds. 2 ± 0.01 g of minced samples were placed into a 15 mL headspace bottle. 1,2-dichlorobenzene (4 μL, 6.42 μg/mL) was added as an internal standard to each sample. The headspace container was sealed with a PTFE diaphragm after being mixed with a vortex. The headspace container was put into a water bath at 55°C for 20 min. The SPME fiber was inserted into a sealed extraction bottle and left on top of the sample for adsorption. The samples were extracted for 30 min and immediately transferred to the GC inlet for 5 min of desorption at 250 °C. The GC conditions were as follows: helium was used as the carrier gas at a flow rate of 1.8 mL/min. The front inlet temperature was 250°C, with a solvent delay of 3 min. The oven temperature was maintained at 40°C for 3 min, ramped to 90°C at a rate of 5 °C/min, then ramped to 230°C at a rate of 8°C/min, and held at 230°C for 10 min. The volatile components from the capillary column were separated into the mass spectrometer (MS) at a ratio of 1:1 (v/v). The MS source was set at 230°C. MS fragmentation was observed in electron-impact (EI) mode (ionization energy of 70 eV) with a full-scan collection range of 20–350 m/z. Compounds were identified based on the NIST 14 database, retention indices (RI) with reference values and authentic volatile standards. The linear retention index values (LRIs) were calculated with a formula by Liu et al. (23).

#### Quantitation and OAVs analysis of aroma compounds

The content of aroma compounds was determined using a 5-point external standard curve. Prior to quantitation analysis, the deodorized matrix was prepared to eliminate the influence of the matrix effect according to a previous method (24), with several modifications. Briefly, diethyl ether and n-pentane were added into the beef (diethylether-n-pentane-beef puree ratio of 2:1:1, m/m/m). After shaking for 12 h, the organic solvent was extracted 5 times. The samples were then frozen in an FD-1A-50 freeze-dryer using liquid nitrogen (Shanghai Zheng-Qiao Science Instrument Plant, Ltd., Beijing, China) at−50°C for 24 h. The concentration of each aroma compound was divided by the reported odor threshold to get the OAVs (25). The contribution rate was the OAVs ratio of each odorant to all odorants.

### Water analysis

The content of moisture in beef samples was determined by DHG-9213A Electric Blast Drying Oven (Jinghong Test Equipment Co., Ltd., Shanghai, China). The sample was dried at 105°C to a constant weight and the mass difference before and after drying was calculated to obtain the moisture content. The moisture distribution of the roasted beef was characterized by an NMI20-NMR analyzer (Niumag Co., Ltd., Shanghai, China) based on the method described by Li et al. (26). Typical pulse parameters were similar to those described by Li et al. (26). The spin-spin relaxation time (*T*_2_) was measured using the Carr–Purcell–Meiboom–Gill sequence with the following parameters: SF = 18 MHz; O1 = 3 82.241 65 Hz; TW = 3000 ms; RFD = 0.2 ms; RG1 = 20 db; P2 = 33 μs; TE = 0.251; NECH = 5000; SW = 100; DRG1 = 3; NS = 8. Three relaxation times (*T*_21_, *T*_22_ and *T*_23_) and their corresponding relaxation signal components (*M*_21_, *M*_22_ and *M*_23_) were recorded (27).

### Sensory evaluation

The sensory evaluation was performed according to previous reports (28, 29). A total of 10 sensory panelists were screened and chosen based on GB/T 16291.1-2012. ISO 4121:2003 and GB/T 29604-2013 guidelines were used to train all panelists. Sensory analysis was performed in an odor-free room at 25 ± 1°C. Sample blocks were put into a 20-mL glass bottle and heated in a water bath at 70 °C until the core temperature reached 35°C. Six aroma attributes, including meaty, fatty, roasty, grassy, sweet and total odors were selected to evaluate the aroma quality of roasted beef. The sensory evaluation panel evaluated the sensory properties of roasted beef on a ten-point scale (8–10: very strong, 6–8: strong, 4–6: medium, 2–4: weak, 0–2: very weak). To avoid odor interaction between samples, panelists were required to take 30 s break.

### Statistical analysis

Results were expressed as the means ± standard deviation. Data were analyzed by ANOVA, followed by Duncan's multiple range test (*P* < 0.05) using SPSS 19.0 software (IBMCorporation, USA). The graphs were made by Origin 18C software, and R software and MetaboAnalyst 4.0 were used to plot and combine figures.

## Results and analysis

### Sensory evaluation

**Figure 1:**
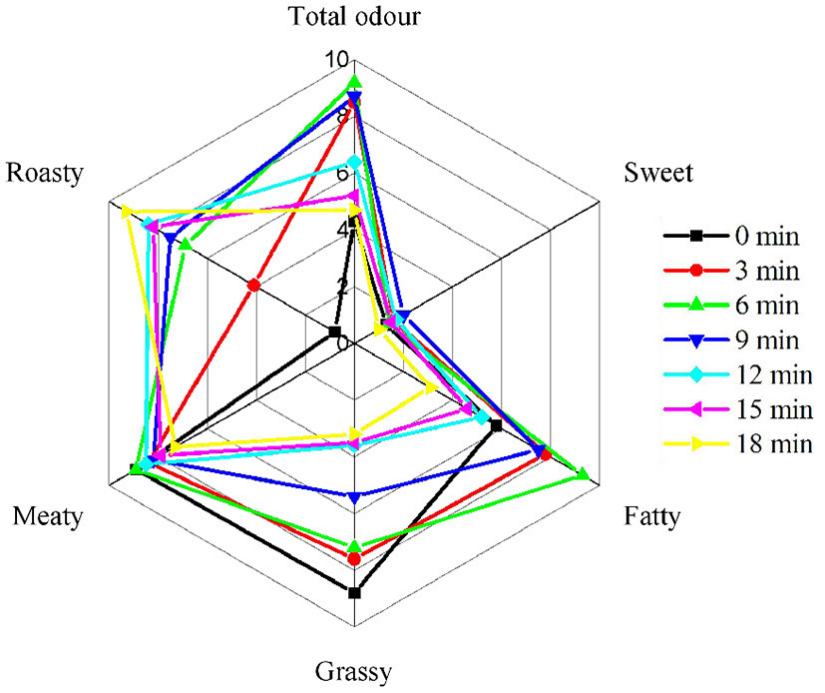
Flavor profiles of roasted beef samples by sensory evaluation.

The purpose of sensory evaluation was to characterize the different flavor profiles of samples. As shown in Figure 1, the beef roasted for 6 min showed significantly high scores for total aroma (*P* < 0.05), indicating that a lot of aroma compounds were generated in the beef. It was worth noting that the sweet aroma of roasted beef showed low scores in all stages, while the meaty aroma exhibited high scores in all stages. In addition, the beef roasted for 18 min gained high scores of roasty aroma, while the raw meat gained high scores of grassy aroma. Moreover, high scores of fatty aroma were observed in the beef roasted for 6 min. However, what aroma compounds were produced by the beef at different roasting times needs further analysis.

### Changes in the color value of beef

The color of the cooked meat provides important information about the quality, flavor and safety of the meat products (30, 31). During the roasting process, the L^*^ values of the beef significantly increased and subsequently decreased after 3 min of roasting (Table 1). Similar results were also reported for the infrared heating (grilling) of fish (32, 33), convective roasting of chicken breast meat (34) or microwave cooking of beef meat (35). Meanwhile, the a^*^ values decreased significantly in the samples roasted for 0–3 min and then kept stable, and the b^*^ values increased in the samples roasted for 0–9 min and then decreased at the end stage of roasting. The ΔE values of the beef were significantly increased (0–14.83) in the samples roasted for 0–3 min, then became stable (13.52–14.83) in the samples roasted for 3–12 min, and finally significantly increased (13.85–18.91) in the samples roasted for 12–18 min.

**Table 1:** Lightness value (L^*^), redness value (a^*^) and yellowness value (b^*^) of the roasted beef.

**Roasting time (min)**	**0**	**3**	**6**	**9**	**12**	**15**	**18**
L*	34.78 ± 0.46c	42.70 ± 1.15a	37.15 ± 1.61b	34.57 ± 1.91c	27.63 ± 2.53d	24.49 ± 0.67e	20.20 ± 0.53f
a*	21.22 ± 0.95a	11.35 ± 0.71b	11.51 ± 1.57b	10.02 ± 1.64bc	11.70 ± 0.33b	8.95 ± 0.93c	9.20 ± 1.42c
b*	4.33 ± 0.51a	12.07 ± 0.60d	11.89 ± 0.99c	12.90 ± 1.10b	9.63 ± 0.99b	9.42 ± 1.05a	3.64 ± 0.57a
Δ E	0.00 ± 0.00f	14.83 ± 0.82c	13.52 ± 1.03de	14.10 ± 1.21cd	13.85 ± 0.54d	16.77 ± 0.87b	18.91 ± 0.75a

### Aroma compounds

#### HS-SPME-GC-MS analysis

**Figure 2:**
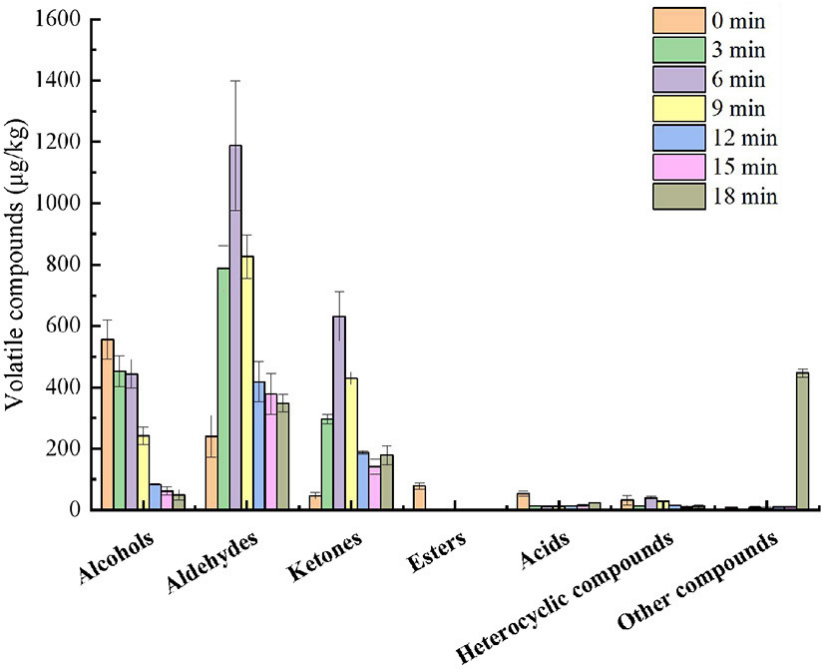
Categories and concentrations of the major volatile compounds in the roasted beef.

Aroma compounds of the beef were extracted by SPME from different roasting times. As shown in Table 2 and Figure 2, a total of 47 odorants were identified and these compounds were classified into seven chemical classes: 14 alcohols, 18 aldehydes, 6 ketones, 1 esters, 3 acids, 4 heterocyclic compounds and 1 other compound. A total of 37, 38, 33, 33, 34, 32, and 28 volatile compounds were identified in the beef at 0, 3, 6, 9, 12, 15, and 18 min, respectively. In the raw meat, methyl ester hexanoic acid, [*Z*]-2-penten-1-ol and [*E, E*]-2,4-non-adienal were identified, whereas they disappeared after roasting for 3 min. The concentrations of 1-penten-3-ol, 1-hexanol, [*E*]-2-octen-1-ol, 1-octen-3-ol, 1-nonanol, [*E*]-2-octenal, [*E, E*]-2,4-decadienal, [*Z*]-6,10-dimethyl-5,9-undecadien-2-one, non-anoic acid and hexanoic acid in the raw meat were significantly higher (*P* < 0.05) than those in the other beef samples roasted for 3–18 min. In contrast, benzaldehyde, heptanal and 2,3-octanedione were observed in the beef roasted for 3 min. The beef roasted for 6 min had the highest concentrations of 1-pentanol, hexanal, heptanal, octanal, non-anal, decanal, dodecanal, tridecanal, tradecanal, pentadecanal, hexadecanal, 2,3-octanedione, nona-3,5-dien-2-one and 2-pentyl-furan. However, the beef roasted for 18 min had the highest concentrations of 2,3-butanediol, 2-furanmethanol, 2-ethyl-1-hexanol, benzaldehyde, acetoin, 6-methyl-5-hepten-2-one and toluene. Interestingly, all pyrazines and piperazines appeared in the samples roasted for 12–18 min, such as 1,4-dimethylpiperazine, 2,5-dimethylpyrazine and 2-ethyl-6-methylpyrazine.

**Table 2:** The concentrations of volatile compounds in the roasted beef.

**Compounds**	**LRIs**		**Aroma content (**μ**g/kg)**						
	**Literature**	**Calculated**	**0 min**	**3 min**	**6 min**	**9 min**	**12 min**	**15 min**	**18 min**
1-Butanol	1,589	1,587	1.30 ± 0.21^a^	0.82 ± 0.11^b^	—	—	0.29 ± 0.02^c^	—	—
2,3-butanediol	1,583	1,585	—	—	—	—	1.78 ± 0.17^c^	2.34 ± 0.73^b^	3.95 ± 0.64^a^
2-Furanmethanol	1,655	1,637	—	—	—	—	—	—	1.17 ± 0.09^a^
1-Penten-3-ol	-	1,317	14.24 ± 1.24^a^	8.18 ± 1.66^c^	10.91 ± 0.99^b^	6.40 ± 1.14^d^	—	—	—
[*Z*]-2-penten-1-ol	1,334	1,357	3.84 ± 1.00^a^	—	—	—	—	—	—
1-Pentanol	1,261	1,263	71.71 ± 9.26^ab^	58.18 ± 9.79^c^	77.25 ± 11.61^a^	40.24 ± 7.80^d^	14.72 ± 1.14^e^	12.14 ± 0.58^f^	6.15 ± 2.16^g^
1-Hexanol	1,359	1,357	71.02 ± 10.85^a^	31.92 ± 5.26^b^	22.53 ± 1.54^c^	10.88 ± 2.25^d^	5.14 ± 0.03^f^	4.58 ± 0.53^g^	6.18 ± 0.84^e^
1-Heptanol	1,462	1,360	13.78 ± 4.14^bc^	20.63 ± 2.53^a^	14.02 ± 0.95^b^	7.44 ± 0.87^d^	3.53 ± 0.16^e^	2.87 ± 0.11^f^	1.63 ± 0.24^g^
1-Octanol	1,573	1,577	28.53 ± 9.69^bc^	42.70 ± 5.49^a^	25.4 ± 0.28^c^	13.73 ± 0.25^d^	7.43 ± 0.24^e^	5.72 ± 2.33^g^	6.32 ± 0.09^fg^
[*E*]-2-octen-1-ol	1,622	1,625	34.84 ± 12.88^a^	21.28 ± 2.30^b^	14.11 ± 0.30^c^	6.58 ± 0.98^d^	—	—	—
1-Octen-3-ol	1,456	1,459	313.89 ± 12.84^a^	264.74 ± 2.59^c^	279.37 ± 10.67^b^	156.78 ± 16.02^d^	49.14 ± 0.76^e^	33.17 ± 5.16^f^	21.21 ± 7.66^g^
2-Ethyl-1-hexanol	1,499	1,477	—	2.11 ± 0.40^a^	—	—	1.43 ± 0.01^c^	1.63 ± 0.61^bc^	2.12 ± 0.34^a^
2-Nonen-1-ol	-	1,771	0.37 ± 0.15^d^	0.40 ± 0.09^d^	0.51 ± 0.06^b^	0.46 ± 0.08^c^	0.46 ± 0.05^c^	—	0.59 ± 0.13^a^
1-Nonanol	-	1,568	2.98 ± 0.93^a^	1.96 ± 0.21^b^	—	—	—	—	—
(*E*)-2-Hexenal	1,196	1,192	1.73 ± 0.45^a^	0.45 ± 0.26^b^	—	—	—	—	—
Hexanal	1,064	1,061	122.67 ± 11.94^f^	513.72 ± 57.48^bc^	811.42 ± 174.10^a^	559.92 ± 58.80^b^	240.7 ± 56.85^d^	242.43 ± 28.05^d^	196.56 ± 14.42^e^
Benzaldehyde	1,534	1,537	—	6.48 ± 0.81^e^	10.44 ± 2.97^d^	10.90 ± 0.10^d^	16.02 ± 0.78^b^	14.62 ± 3.88^bc^	24.28 ± 2.57^a^
[*E*]-2-heptenal	1,331	1,327	5.94 ± 3.25^ab^	4.14 ± 1.36^b^	1.99 ± 0.67^cd^	1.68 ± 0.23^d^	—	—	—
Heptanal	1188	1189	—	41.70 ± 4.73^c^	69.69 ± 8.48^a^	52.27 ± 5.36^b^	30.16 ± 3.89^d^	22.96 ± 6.53^e^	17.22 ± 1.84^f^
Octanal	1,291	1,294	8.64 ± 3.25^g^	50.92 ± 0.56^c^	70.46 ± 7.09^a^	56.28 ± 2.82^b^	32.75 ± 1.12^d^	27.66 ± 2.67^e^	22.83 ± 3.12^f^
[*E*]-2-octenal	1,434	1,437	6.35 ± 2.70^a^	4.27 ± 0.07^b^	3.95 ± 0.07^c^	2.23 ± 0.17^d^	1.12 ± 0.04^e^	1.00 ± 0.01^f^	—
[*E, E*]-2,4-nonadienal	1,714	1,716	6.67 ± 4.00^a^	—	—	—	—	—	—
nonanal	1,396	1,394	44.84 ± 8.50^g^	138.39 ± 4.67^b^	179.55 ± 11.75^a^	126.45 ± 0.16^c^	83.58 ± 2.26^d^	60.27 ± 22.67^f^	81.84 ± 0.75^e^
[*E, E*]-2,4-Decadienal	1,826	1,822	17.3 ± 1.95^a^	5.08 ± 0.64^b^	4.35 ± 0.56^c^	2.90 ± 0.19^d^	1.36 ± 0.05^e^	1.29 ± 0.06^f^	1.12 ± 0.23^g^
Decanal	1,504	1,505	4.33 ± 1.32^cd^	4.82 ± 0.33^c^	6.78 ± 0.65^a^	5.10 ± 1.09^b^	4.45 ± 0.65^cd^	3.12 ± 0.71^e^	4.84 ± 0.34^c^
2-Undecenal	1,741	1,728	1.60 ± 0.37^b^	2.22 ± 0.28^a^	1.14 ± 0.08^c^	0.89 ± 0.01^d^	0.68 ± 0.03^f^	0.73 ± 0.31^e^	—
Undecanal	-	1,306	0.53 ± 0.15^c^	0.77 ± 0.03^a^	—	0.68 ± 0.17^ab^	0.30 ± 0.05^d^	—	0.18 ± 0.14^e^
Dodecanal	-	1,435	1.19 ± 0.30^b^	1.21 ± 0.14^b^	1.56 ± 0.11^a^	0.61 ± 0.05^c^	0.69 ± 0.26c	0.25 ± 0.04^d^	—
Tridecanal	-	1572	3.67 ± 1.56^b^	3.36 ± 0.46^b^	5.07 ± 0.09^a^	—	1.62 ± 0.04^c^	—	—
Tetradecanal	-	1,728	4.35 ± 2.16^b^	3.40 ± 0.73^c^	6.19 ± 0.49^a^	1.63 ± 0.03^d^	1.12 ± 0.20^e^	—	—
Pentadecanal	-	1,953	8.90 ± 5.88^b^	5.15 ± 0.74^c^	11.6 ± 1.95^a^	3.20 ± 1.94^d^	1.91 ± 0.11^e^	1.15 ± 0.24^f^	—
Hexadecanal	-	2,214	1.50 ± 1.04^c^	1.44 ± 0.12^c^	3.69 ± 1.10^a^	1.54 ± 0.18^c^	2.49 ± 0.03^b^	2.59 ± 1.03^b^	—
Acetoin	-	955	38.21 ± 7.31^d^	55.37 ± 2.13^b^	46.10 ± 14.60^c^	38.46 ± 0.21^d^	49.12 ± 3.68^c^	56.86 ± 3.06^b^	111.63 ± 12.28^a^
6-Methyl-5-hepten-2-one	1,342	1,328	1.54 ± 0.46^b^	1.10 ± 0.29^c^	1.66 ± 0.38^b^	0.85 ± 0.10^d^	1.14 ± 0.09^c^	1.14 ± 0.29^c^	2.98 ± 1.19^a^
2,3-Octanedione	1,324	1,318	—	236.07 ± 11.84^c^	575.59 ± 64.99^a^	387.78 ± 20.64^b^	135.35 ± 1.38^d^	82.46 ± 19.49^e^	46.42 ± 16.21^f^
Nona-3,5-dien-2-one	-	1,544	3.57 ± 2.31^ab^	2.21 ± 0.30^c^	4.42 ± 0.07^a^	0.92 ± 0.07^d^	—	—	—
2,3-Dihydro-3,5-dihydroxy-6-methyl-4H-pyran-4-one	1,143	1,147	—	—	—	—	—	1.17 ± 0.72^b^	17.15 ± 1.14^a^
[*Z*]-6,10-dimethyl-5,9-undecadien-2-one	-	1,739	3.57 ± 0.82^a^	2.34 ± 0.30^c^	3.21 ± 0.53^b^	1.85 ± 0.11^d^	1.73 ± 0.19^d^	1.04 ± 0.26^e^	1.70 ± 0.15^d^
Hexanoic acid methyl ester	1,189	1,190	78.27 ± 10.55^a^	—	—	—	—	—	—
Acetic acid	1,460	1,459	4.88 ± 0.08^e^	5.15 ± 0.38^d^	5.12 ± 2.13^d^	5.31 ± 0.16^d^	6.00 ± 0.90^c^	10.82 ± 0.06^b^	21.24 ± 0.01^a^
Hexanoic acid	1,854	1,855	46.94 ± 4.45^a^	7.33 ± 0.60^b^	6.34 ± 0.15^d^	5.21 ± 0.30^e^	7.09 ± 0.79^bc^	4.24 ± 0.97^f^	3.41 ± 0.16^g^
Nonanoic acid	-	1,929	1.85 ± 0.99^a^	1.06 ± 0.13^bc^	1.14 ± 0.30^b^	1.01 ± 0.19^bc^	1.02 ± 0.07^c^	0.88 ± 0.00^d^	—
2-Pentylfuran	1,230	1,232	32.27 ± 5.50^b^	13.89 ± 0.35^e^	40.29 ± 4.33^a^	29.78 ± 1.83^bc^	15.70 ± 1.87^d^	7.28 ± 3.45^f^	8.20 ± 4.26^f^
1,4-Dimethylpiperazine	-	1,359	—	—	—	—	—	0.92 ± 0.49^b^	3.32 ± 1.18^a^
2,5-Dimethylpyrazine	1,287	1,391	—	—	—	—	—	2.28 ± 0.51^b^	4.17 ± 0.23^a^
2-Ethyl-6-methylpyrazine	1,368	1,429	—	—	—	—	0.35 ± 0.00^c^	0.58 ± 0.09^b^	0.67 ± 0.14^a^
Toluene	-	787	9.32 ± 0.72^b^	4.55 ± 0.77^d^	8.80 ± 3.37^b^	5.87 ± 3.87^cd^	9.48 ± 0.63^b^	9.75 ± 0.71^b^	446.34 ± 13.04^a^

In all stages, aldehydes and alcohols had the highest concentrations in either raw or roasted beef. Hexanal, heptanal, octanal and nonanal were the major aldehydes, with hexanal having the highest value. 1-pentanol, 1-hexanol, 1-heptanol, 1-octanol and 1-octen-3-ol were the main alcohols in the roasted beef. Compared to the raw meat, the concentrations of alcohols in the roasted meat decreased with the increase of roasting times. While the levels of aldehydes, ketones and heterocyclic compounds significantly increased in the roasted beef after roasting for 3–6 min (*P* < 0.05) and subsequently decreased after roasting for 9 min. In particular, 1-pentanol (77.25 μg/kg), hexanal (811.42 μg/kg), heptanal (69.69 μg/kg), octanal (70.46 μg/kg), nonanal (179.55 μg/kg), 2,3-octanedione (575.59 μg/kg) and 2-pentylfuran (40.29 μg/kg) predominantly contributed to the aroma in the beef roasted for 6 min.

#### Key aroma compounds in roasted beef

**Figure 3:**
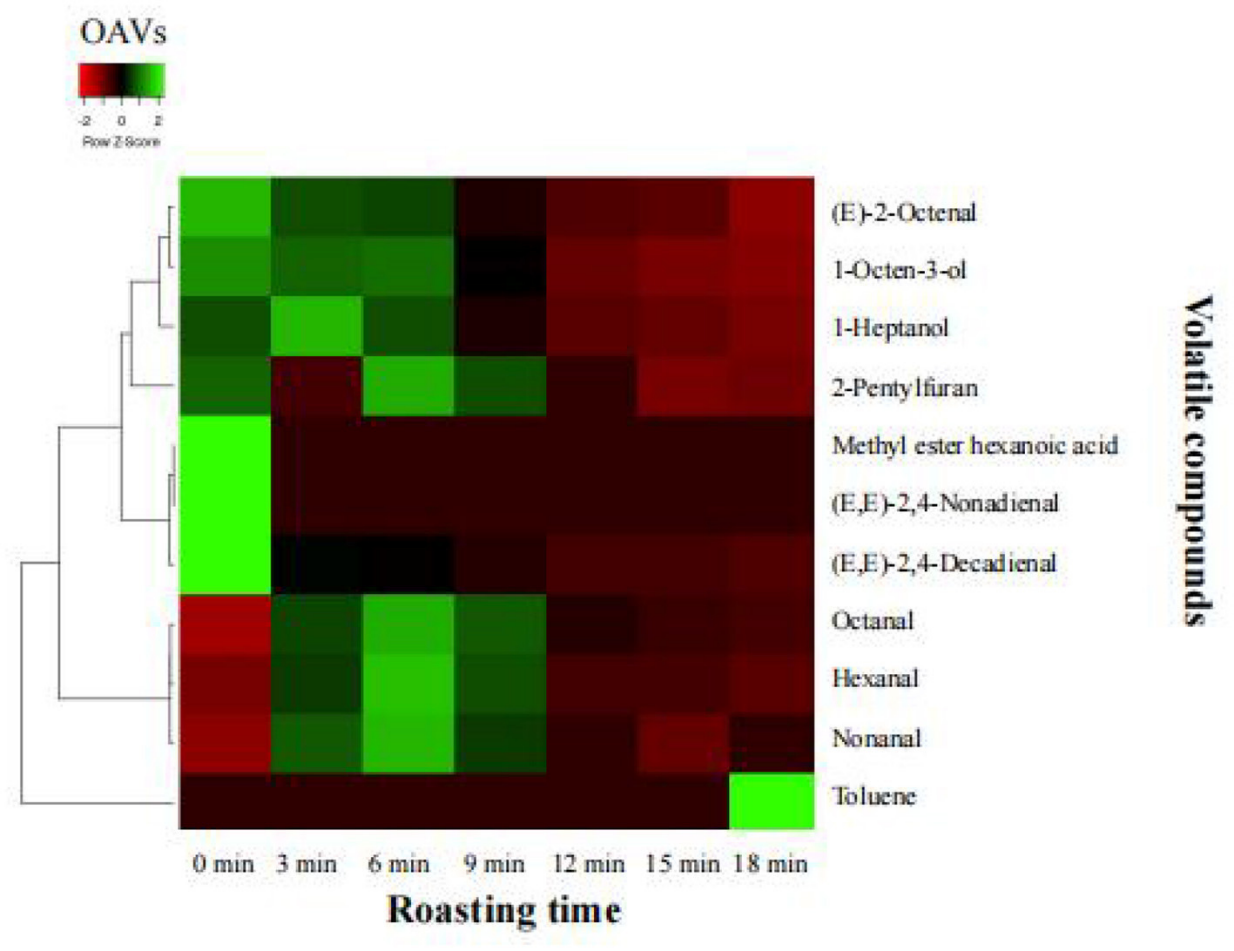
Changes of OAVs of key aroma compounds (OAVs >1) in the roasted beef during roasting.

Aroma compounds (OAVs >1) played a vital role in the aroma expression of the samples. To better understand the importance of each aroma compound, the OAVs and contribution rates were calculated. As shown in Figure 3, a total of 11 aroma compounds were found to be the key odorants in the roasted beef, including 1-heptanol, 1-octen-3-ol, hexanal, octanal, (*E*)-2-octenal, (*E, E*)-2,4-nonadienal, nonanal, (*E, E*)-2,4-decadienal, methyl ester hexanoic acid, 2-pentylfuran and toluene. Ten out of 11 odorants played vital roles in aroma expression in the raw meat with OAVs >1, including 1-heptanol, 1-octen-3-ol, hexanal, octanal, (*E*)-2-octenal, (*E, E*)-2,4-nonadienal, nonanal, (*E, E*)-2,4-decadienal, methyl ester hexanoic acid and 2-pentylfuran. Eight out of 11 odorants were initially considered the key odorants in the beef roasted for 6 min with OAVs >1, including 1-heptanol, 1-octen-3-ol, hexanal, octanal, (*E*)-2-octenal, nonanal, (*E, E*)-2,4-decadienal, and 2-pentylfuran. The concentrations and OAVs of 1-octen-3-ol, (*E*)-2-octenal and (*E, E*)-2,4-decadienal decreased significantly (*P* < 0.05) from 0 to 18 min. However, the levels of hexanal, octanal and nonanal increased significantly (*P* < 0.05) from 0 to 6 min, and then their values decreased significantly (*P* < 0.05) from 9 to 18 min. In comparison with the raw meat, 4 key odorants were all detected and were maintained at high levels in the samples roasted for 6 min, among which hexanal (16.23), octanal (100.66), nonanal (179.55), and 2-pentylfuran (6.72) had the highest OAVs. Particularly, 1-octen-3-ol had the highest concentration and OAV (*P* < 0.05) in the raw meat, followed by the samples at 6 min, and the lowest concentration was found in the beef roasted for 18 min. The contribution rate was further used to exhibit the importance of each odorant. 1-octen-3-ol (41.35%), nonanal (26.58%), octanal (14.90%), (*E, E*)-2,4-decadienal (12.88%), hexanal (2.40%) and 2-pentylfuran (0.99%) predominantly contributed to the aroma in the beef roasted for 6 min. Furthermore, raw meat, roasted beef, and the beef that exceeded the roasting time could be discriminated by the concentrations of 1-octen-3-ol, octanal and nonanal.

### The changes of water in the process of beef roasting

NMR spectroscopy, a well-established method for characterizing the state, mobility, and distribution of water in polymer systems, has been widely used (36). In the study, the proton transverse relaxation time (*T*_2_) was used to evaluate the water distribution and properties in myofibrillar proteins of the beef samples during roasting. There are three distinct types of water, *T*_21_, *T*_22_ and *T*_23_. The relaxation signal components of *T*_21_, *T*_22_, and *T*_23_ are represented by *M*_21_, *M*_22_ and *M*_23_, respectively. The water populations reflected the mobility of water fractions from the most tightly bound to the least tightly bound, which were described as bound water (*T*_21_), immobilized water (*T*_22_) and free water (*T*_23_), respectively (37). *T*_21_ (0.01 to 10 ms) is tightly connected with hydrophilic groups in macromolecules (38); *T*_22_ (10–100 ms) is the major water component among the three varieties of water in the muscle and is entrapped in the myofibrillar network or between the thin and thick filaments (39); *T*_23_ (100–1,000 ms) resides between fiber bundles and is easy to lose (40).

**Figure 4:**
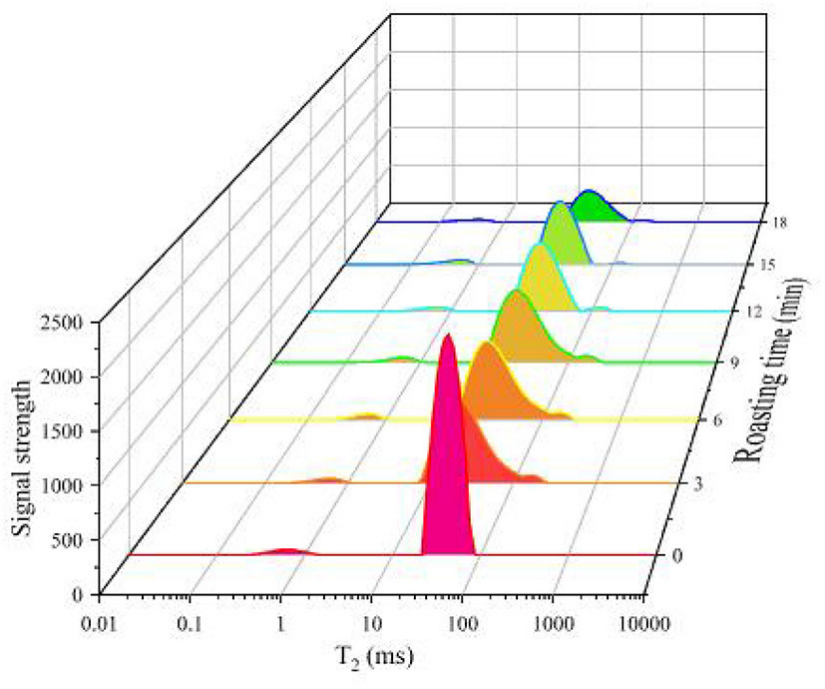
*T*_2_ relaxation time distribution curve of the roasted beef at the different roasting times.

As shown in Table 3 and Figure 4, the total peak area decreased gradually with increasing roasting time, indicating that the moisture content of the samples decreased. The peak positions of *T*_22_ and *T*_23_ shifted to the left, indicating that the fluidity of the water in the samples was decreased and the water with a high degree of freedom migrated to the water with a low degree of freedom.

**Table 3:** Effects of moisture content in the materials on the *T*_2_ relaxation time of roasted beef.

**Roasting time (min)**	**0**	**3**	**6**	**9**	**12**	**15**	**18**
*T*_21_ (ms)	1.45 ± 0.12b	1.81 ± 0.17a	1.15 ± 0.18de	1.16 ± 0.16de	1.25 ± 0.15d	1.22 ± 0.12d	1.31 ± 0.00cd
*T*_22_ (ms)	65.43 ± 0.00c	167.70 ± 0.00a	125.14 ± 0.00b	125.14 ± 0.00b	69.20 ± 0.53c	43.63 ± 0.00d	69.51 ± 0.00c
*T*_23_ (ms)	0.00 ± 0.00e	185.25 ± 30.36a	113.62 ± 0.00d	126.88 ± 22.97bc	113.95 ± 0.00d	135.13 ± 0.00b	128.99 ± 21.28bc
Moisture content (%)	70.94 ± 1.34a	64.01 ± 2.55b	62.96 ± 0.67c	61.55 ± 0.77d	55.00 ± 1.98e	45.27 ± 2.48f	39.10 ± 2.29g
*M*_21_ (%)	3.10 ± 0.14g	3.95 ± 0.49f	4.25 ± 0.52ef	4.51 ± 0.28d	4.96 ± 0.54cd	5.82 ± 0.90ab	5.65 ± 0.14b
*M*_22_ (%)	96.90 ± 0.14a	94.10 ± 0.65b	93.10 ± 0.48c	92.71 ± 0.16d	92.57 ± 0.55d	93.00 ± 0.86e	91.92 ± 0.00f
*M*_23_ (%)	0.00 ± 0.00e	1.95 ± 0.44c	2.65 ± 0.13a	2.78 ± 0.25a	2.46 ± 0.09b	1.17 ± 0.04d	2.43 ± 0.15b

## Discussion

### Sensory evaluation

The aroma is one of the most predominant qualities that affect the product's sensory characteristics. The total concentration of the compounds identified by GC-MS was the highest in the beef roasted for 6 min, especially alcohols and aldehydes. This may be the reason for the high total odor score of the beef roasted for 6 min. In addition, a high score of roasted aroma was observed in the beef roasted for 18 min, which may be related to the heterocyclic compounds generated from the Maillard reaction, like 2,5-dimethylpyrazine and 2-ethyl-6-methylpyrazine (41). Pyrazines, particularly alkylpyrazines, are extensively distributed and are responsible for the nutty, musty, or cocoa aroma odor in the roasted foods (41). The fatty aroma may be related to the linear aldehydes formed by lipid oxidation like hexanal, nonanal (*E, E*)-2,4-nonadienal and octanal. Numerous studies have shown that these compounds have a fatty aroma (7, 24, 42). Alcohols generally have lower thresholds with a green aroma, and large amounts of alcohol were observed in the raw meat.

### Color

In general, the levels of undenatured myoglobin (including oxymyoglobin) and globin haemochromogen influenced the color of the cooked meat (43). However, the roasting process was the primary reason for the changes in the meat color during a high temperature and long time roasting. In this study, the increase of the L^*^ values and ΔE values in the beef roasted for 0–3 min may attribute to the changes in the levels of undenatured myoglobin and globin haemochromogen. The changes in the L^*^value of the beef samples roasted for 3–18 min may affect water content, which was quickly decreased due to evaporation during sustained high-temperature heating (44). The decrease of water content may result in a faster heating rate in the samples given the same heat per unit time, and may also accelerate some reactions, like the Maillard reaction. In the final stage of the Maillard reaction, condensation of carbonyls and amines produces brown-colored high molecular weight compounds, known as melanoidins (browning), resulting in the decrease of the L^*^value (45). This appearance was obvious and could be directly observed by the naked eyes (46). Meanwhile, a decrease in the water content may lead to reduced reflection of light, also decreasing the L^*^value (47). The ΔE values of the beef were significantly increased (*P* < 0.05) in the samples roasted for 12–18 min. This may be explained by a finding showing that the heat-induced unfolding of proteins may play an important role in the process of the Maillard reaction (48). The a^*^ value, the most sensitive parameter of color measurement, reflects red color and color stability (49). The color of the roasted beef changed from light red to pale, with a decrease of a^*^ value. Roasting also significantly increased the b^*^ value of the roasted beef (*P* < 0.05). However, long time heat treatment at high temperatures may result in an unattractive color.

### Aldehydes and alcohols predominantly contributed to the key aroma compounds in the roasted beef

Aldehydes and alcohols, which resulted from lipid oxidation as well as Maillard reactions and Strecker degradation of carbohydrates and amino acids, were the primary aroma compounds in meat products, such as 1-octen-3-ol nonanal, octanal and hexanal (50). The unsaturated fatty acids, like linoleic acid and α-linolenic acid, predominantly contributed to the formation of fatty aldehydes and alcohols such as 1-pentanol, pentanal, 1-hexanol, 1-heptanol, 1-octanol, hexanal, heptanal, decanal, octanal, nonanal, benzaldehyde (*E*)-2-octenal, 1-nonanol, 1-octen-3-ol and (*E*)-2-octen-1-ol (42, 50). In this study, a total of 47 aroma compounds were identified, including 14 alcohols and 18 aldehydes. A total of 11 aroma compounds were selected through OVAs as the key odorants of the roasted beef. However, 8 aldehydes and alcohols out of 11 key odorants were observed, with a high contribution (97.36–99.61%) to the aroma in the roasted beef. Its was also reported that aldehydes were the predominant class of compounds found in the roasted beef, followed by alcohols (51). In the beef roasted for 6 min, 1-octen-3-ol had the highest OAVs (313.89) and contribution rate (41.35%), followed by nonanal, octanal, (*E, E*)-2,4-decadienal and hexanal. Different roasting methods could change the concentrations of the aldehydes and alcohols, but these odors were still the most critical aroma compounds in the roasted beef (52). Furthermore, the concentrations of hexanal, octanal, and 1-octen-3-ol were higher than other compounds in the roasted meat at different aging stages, with the highest OAVs (25). 2-pentylfuran (OAVs >1) was observed in the roasted beef, and may also be a key aroma compound. In addition, harmful compound toluene was observed with OAVs >1 in the beef roasted for 18 min. Different from other key compounds, methyl ester hexanoic acid was only found in the raw meat.

### Aroma contribution of key aroma compounds in roasted beef

The typical meaty, fatty, roasty, grassy and sweet aromas in the roasted beef were mostly produced by the 11 key aroma compounds with OAVs >1. 1-octen-3-ol, a key aroma compound in the roasted beef with mushroom aromas, was enzymatically produced by hydrolyzation and oxidation of the n-3, n-6 polyunsaturated fatty acids during the heating process (24). Octanal (fatty and green aromas), 1-heptanol (floral and green aromas) and nonanal (rose, citrus, and strong fat flavor) were derived from oleic acid oxidation (24, 53–55). (*E, E*)-2,4-decadienal (fruity/sweet orange, sweet melon and fatty/toasted/scallion biscuit aromas) formed by oxidation of n-3 unsaturated fatty acids when heated at 85 and 100 °C (55). (*E, E*)-2,4-nonadienal (toasted and fatty aromas) and (*E*)-2-octenal (grilled meat and peanut cake aromas) were generated from the degradation of linoleic acid at high temperatures (55). Hexanal was a product of linoleic acid oxidation and contributed mainly to fatty and grassy aroma (56). In addition, the ratio of hexanal to nonanal was proposed as an indicator of mutton freshness and overall quality (57). 2-pentyl-furan was responsible for the aromas of earthy and green notes and may be generated by autoxidation and oxidation of n-6 unsaturated fatty acids, such as linoleic acid (56). Toluenes may have no contribution to or negatively affect the aroma characteristics of the roasted beef if too many of these components were present (58). The concentrations of 11 key aroma compounds in the beef decreased during the roasting process, while the levels of aldehydes and 2-pentylfuran were increased in the beef roasted for 0–6 min and produced fatty and meat aroma. The whole aroma of the roasted beef gradually decreased during roasting for 6–18 min. The concentrations of each key aroma compound increased or decreased during the roasting process. The aroma of the roasted beef was dominated by the synergy of key aroma compounds (59).

### Moisture migration of beef during the roasting process

Longer transverse relaxation time is related to higher degrees of freedom, while shorter transverse relaxation time is associated with lower degrees of freedom (60). As shown in Table 3, the value of *T*_21_ in the roasted beef decreased with increasing roasting time (*P* < 0.05). This result suggested that the water in the beef with a high degree of freedom migrated to the water with a low degree of freedom and the water was gradually restricted by the proteins during the roasting procedure (14). The changes of *T*_22_ in the roasted beef were similar to *T*_21_. The decrease in *T*_22_ indicated that the water located in the myofibrillar network of the roasted beef with increasing roasting time may flow into the extra-myofibrillar network space owing to myofibrils contraction (61). The changes in *T*_23_ in beef during the roasting process were not significant (*P* > 0.05).

The percentages of peak areas (*M*_21_, *M*_22_, and *M*_23_) corresponded to *T*_2_ relaxation time. The *M*_21_ and *M*_22_ in the roasted beef with the increase of roasting time were significantly increased and decreased, respectively (*P* < 0.05). No significant (*P* > 0.05) differences in *M*_23_ of the roasted beef samples were observed. The decrease in *M*_22_ indicated that the immobilized water entrapped in the myofibrillar network was transformed into free water located in the intercellular space during the roasting process. This result was similar to a previous report showing that the water amounts corresponding to the *T*_22_ domain were redistributed into the intercellular space, resulting in a decrease in the water-holding capacity of samples (62).

### Correlation between color, moisture and key aroma compounds

**Figure 5:**
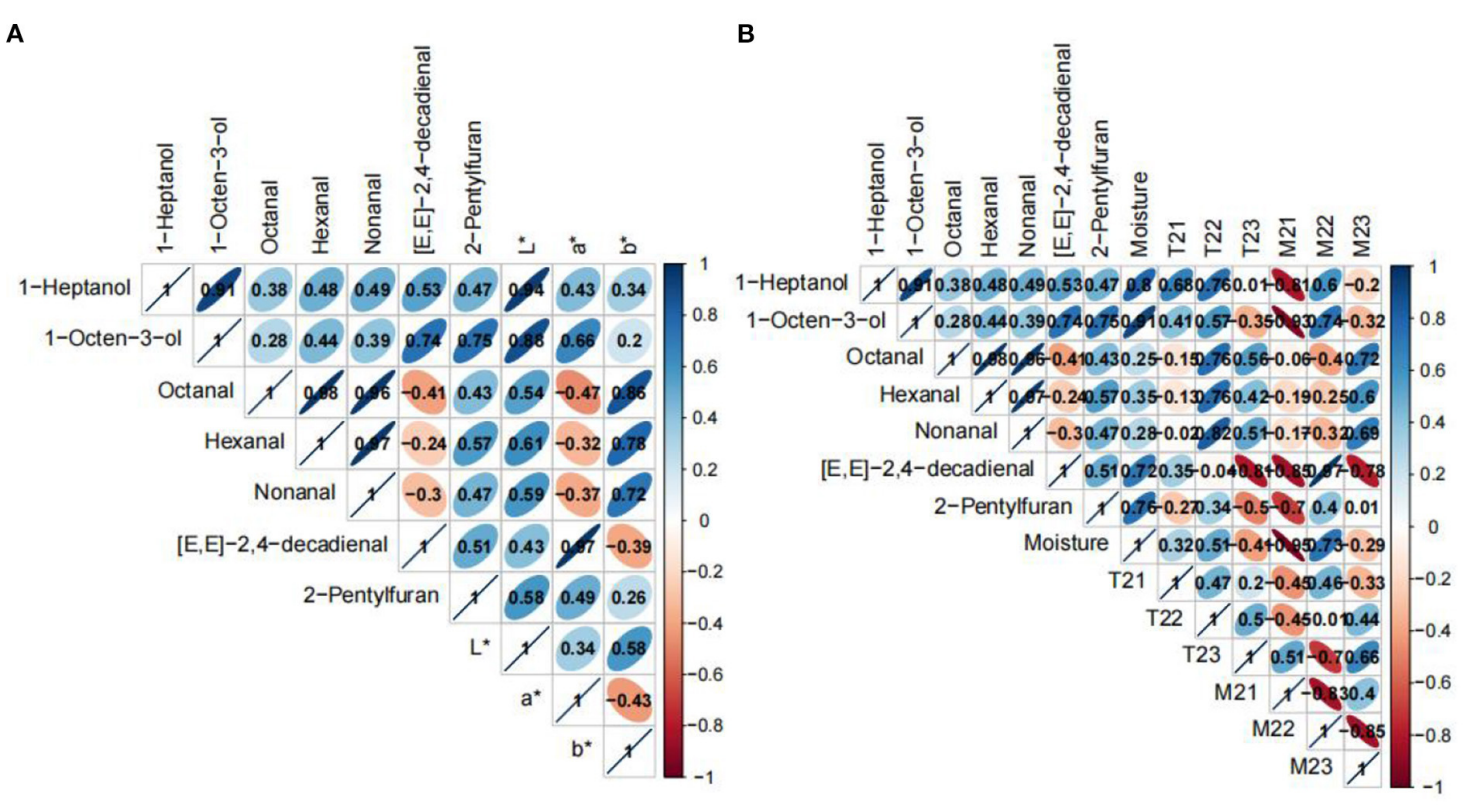
Correlation analysis among color value **(A)**, water content **(B)** and key aroma compounds.

Based on OAVs analysis, 7 key aroma compounds present in all samples were selected to correlate with color and water (Figure 5). The water content was negatively correlated with all the key aroma compounds, while *M*_21_ was positively correlated with all the key aroma compounds. It was reported that the moisture was easily lost by evaporation during heating at high temperatures for a prolonged time (63). The heat that causes water loss in the samples may also accelerate the generation of aroma compounds (64). *M*_21_ represents bound water, and it's not easy to lose during the roasting process because the water is tightly associated with hydrophilic groups in macromolecules. *T*_21_ was negatively correlated with 1-heptanol, 1-octen-3-ol and [*E, E*]-2,4-decadienal. *T*_22_ was negatively correlated with all the key aroma compounds, except [*E, E*]-2,4-decadienal. *T*_23_ was positively correlated with [*E, E*]-2,4-decadienal and 1-octen-3-ol. A study showed that water-holding capacity had a clear link with *T*_22_ and *T*_23_ populations (28). *M*_22_ was negatively correlated with [*E, E*]-2,4-decadienal, 1-octen-3-ol, 1-heptanol and 2-pentylfuran. *M*_23_ was negatively correlated with nonanal, hexanal and octanal. L^*^ was negatively correlated with all the key aroma compounds. a^*^ was negatively correlated with [*E, E*]-2,4-decadienal, 1-octen-3-ol and 1-heptanol. b^*^ was negatively correlated with nonanal, hexanal and octanal. The difference in the L^*^value among the samples may be attributed to different moisture contents in the roasted beef samples. The samples with a lower water content possessed lower L^*^ values.

## Conclusions

The present work revealed the law of water distribution and the changes in aroma compounds in the roasted beef under the electric roasting process. A total of 47 volatile compounds were identified and 11 key aroma compounds were selected. In all stages, aldehydes and alcohols were the key aroma compounds. The fluidity of the water in the beef during the roasting process decreased, and the water with a high degree of freedom migrated to the water with a low degree of freedom. The moisture content and L^*^ value were negatively correlated with the key aroma compounds, while *M*_21_ was positively correlated with the key aroma compounds. The color and water content are important parameters to evaluate the quality of the roasted beef. Thus, we hope that a predictive model between indicators, such as color and moisture content and the degree of roasting can be established in the future. This predictive model may be used to control the color and water level of the beef during the process of electric roasting, and at the same time ensure the safety of the final product. Furthermore, the roasting process can be optimized through this predictive model to make the production processes intelligent and to obtain the product with the highest quality for the consumers.

## Data availability statement

The original contributions presented in the study are included in the article/supplementary material, further inquiries can be directed to the corresponding author/s.

## Author contributions

Y-RW: resources, software, investigation, methodology, writing—original draft, and writing—review and editing. S-LW: conceptualization and supervision. R-ML and Y-RW: validation and visualization. All authors contributed to the article and approved the submitted version.

## Funding

The State Key Research and Development Plan (2018YFD0400101), the Natural Science Foundation of China (31660484), and the Key Research and Development Plan of Ningxia Hui Autonomous Region (2019BEH03002) provided financial assistance for this work.

## Conflict of interest

The authors declare that the research was conducted in the absence of any commercial or financial relationships that could be construed as a potential conflict of interest.

## Publisher's note

All claims expressed in this article are solely those of the authors and do not necessarily represent those of their affiliated organizations, or those of the publisher, the editors and the reviewers. Any product that may be evaluated in this article, or claim that may be made by its manufacturer, is not guaranteed or endorsed by the publisher.

## Abbreviations

GC-MS: Gas chromatography-mass spectrometry
LF-NMR: Low field-nuclear magnetic resonance
LRIs: linear retention index values
OAVs: Odor activity values.
